# Rapid Diagnostic Tests and Severity of Illness in Pandemic (H1N1) 2009, Taiwan

**DOI:** 10.3201/eid1607.100105

**Published:** 2010-07

**Authors:** Tsui-Mai Kao, Un-In Wu, Yee-Chun Chen

**Affiliations:** Author affiliations: National Taiwan University Hospital, Taipei, Taiwan; and National Taiwan University College of Medicine, Taipei

**Keywords:** Rapid diagnostic tests, viruses, respiratory infections, severity of illness, pandemic (H1N1) 2009, influenza, Taiwan, letter

**To the Editor:** The recent pandemic (H1N1) 2009 (*1*) demonstrates the need for more sensitive rapid diagnostic tests (RDTs) to distinguish between influenza and other respiratory viruses, enhance influenza surveillance, and institute early antiviral therapy for patients who are severely ill or at high risk (*2*). In anticipation of the global spread of pandemic (H1N1) 2009, on August 15, 2009, the government of Taiwan began performing RDTs at clinics and hospitals for patients with influenza-like illness. This initiative was based in part on the notion that patients with higher viral loads would be more likely to have a positive RDT result and more severe disease. We report that RDTs may have paradoxically lower sensitivity for pandemic (H1N1) 2009 virus for patients with respiratory failure requiring mechanical ventilation, extracorporeal membrane oxygenation (ECMO), or both than for those without respiratory failure.

National Taiwan University Hospital is a 2,200-bed teaching hospital in Taiwan. This hospital provides primary and tertiary care and ECMO. All patients admitted with presumed severe influenza were immediately treated with oseltamivir during the 2009 pandemic. From July 25 through December 28, 2009, we studied 20 patients with confirmed disease and 3 adult patients with suspected disease who met the US Centers for Disease Control and Prevention case definitions for pandemic (H1N1) 2009 (*3*).

An RDT (QuickVue A+B; Quidel, San Diego, CA, USA) was performed by using nasopharyngeal swab specimens. Genetic material specific for pandemic (H1N1) 2009 viruses was detected in nasopharygeal or throat swab specimens by real-time reverse transcription–PCR at the Centers for Disease Control and Prevention, Taiwan (*4*). Demographic and clinical characteristics of the 23 patients are shown in the Table.

**Table:** Characteristics of 23 hospitalized patients with confirmed (n = 20) and suspected (n = 3) pandemic (H1N1) 2009, Taiwan*

Characteristic	No mechanical ventilation (n = 8)	Mechanical ventilation	
		Non-ECMO (n = 7)	ECMO (n = 8)
Age, y			
Mean ± SD	39.9 ± 12.8	44.4 ± 19.5	34.6 ± 15.0
>65, no. (%)	0	1 (14.3)	0
Male sex, no. (%)	4 (50)	2 (28.6)	4 (50)
BMI, median (IQR)	24.2 (21.2–31.3)	19.4 (17.3–22.6)	27.6(21.9–33.6)
No. (%) with other diseases	4 (50)	7 (100)	3 (37.5)†
Highest SOFA score, mean ± SD‡	0.89 ± 0.93§	9.7 ± 5.6	10.5 ± 4.0
SOFA score >4, no. (%)	0§	7 (100)	8 (100)
APACHE II score, mean ± SD¶	1. 6 ± 2.5§	17 ± 6.9	19.6 ± 3.8
APACHE II score >15, no. (%)	0§	5 (71.4)	8 (100)
Secondary bacterial infection			
* Streptococcus pneumoniae*	1	0	1
β-Streptococci, non-A, B, D	0	0	1
* Klebsiella pneumoniae*	0	1	0
Duration from illness onset to first medical access, d, median (IQR)	2 (1–2.2)	3 (2.5–3)	2 (2–3)
RDT sensitivity, n/N (%)	8/8 (100) §	3/7 (42.9)	1/8 (12.5)
Duration from illness onset to antiviral therapy, d, median (IQR)	4 (3.8–5.8)	6 (4.5–6)	6.5 (5.5–7.2)
Duration of ICU stay, d, median (IQR)	0 (0–2)§	15 (11.5–27.5)	49.5 (22.8–56.2)
Length of hospital stay, d, median (IQR)	5.5 (4–9.2)§	27 (21.5–54.2)	55.5 (30.2–71.2)
28-day mortality rate, no. (%)	0	1 (14.3)	0
Complications			
Ventilator dependent	0	3	3
Hemodialysis	0	0	1

*ECMO, extracorporeal membrane oxygenation; BMI, body mass index; IQR, interquartile range; SOFA; Sequential Organ Failure Assessment; APACHE, Acute Physiology and Chronic Health Evaluation; RDT, rapid diagnostic test; ICU, intensive care unit. †A 49-year-old man who had a renal transplant, a 17-year-old woman with congenital heart disease, and a 64-year-old man with diabetes and hypertension. ‡Range 0–24. Higher values indicate more severe disease. §p<0.05 for 8 patients without mechanical ventilation vs. 15 patients with mechanical ventilation with and without ECMO support. ¶Range 0–71. Higher values indicate more severe disease.

Severity of illness was assessed within 24 hours of admission by determining the Acute Physiology and Chronic Health Evaluation II score (*5*). The highest Sequential Organ Failure Assessment score was calculated to predict outcome of critically ill patients during their stay in the intensive care unit (*6*). The Student *t* test was used to assess continuous variables, and χ^2^ or Fisher exact tests were used to assess discrete variables. A p value <0.05 was considered significant. Statistical analyses were performed by using SAS software version 9.1 (SAS Institute, Cary, NC, USA).

There were no differences in age, sex, body mass index, underlying diseases, or occurrence of secondary bacterial infection between patients who received mechanical ventilation (n = 15) and those who did not (n = 8). There were no significant differences between the 2 groups in the median number of days from onset of illness to access to medical care. Patients receiving mechanical ventilation had higher severity-of-illness scores and longer times in the intensive care unit and the hospital. Sensitivity of the RDT was 100% for patients who did not receive mechanical ventilation and 26.7% for those who did (p<0.0001).

Median age of the 8 patients who received ECMO was 31 years. Only 3 patients had underlying diseases. RDT results were positive for only 1 of these patients. Five patients were tested by RDT more than one time before transfer or hospitalization. The interval from onset of illness to the first RDT was 1 d for 1 patient, 2 d for 4 patients, 3 d for 2 patients, and 6 d for 1 patient. Failure of the RDT to detect influenza was associated with a delay of >5 d in instituting antiviral therapy for 6 of 8 patients who received ECMO. However, ECMO was stopped for 7 patients who were discharged from hospital after a median duration of 23 d (interquartile range 11.5–54 d) of ECMO.

This report demonstrates an apparently paradoxical inverse relationship between a positive RDT result and severity of illness among patients with pandemic (H1N1) 2009. This observation cannot be explained by differences in the time to access to medical care, performance of RDT (*7*), or prior antiviral therapy. Variants of pandemic (H1N1) 2009 virus may preferentially infect the lower respiratory tract in certain hosts (*8*). Invasive properties of pandemic (H1N1) 2009 virus and severity of illness may be more closely related to heterogeneity in host immunity than to viral load (*9*). US Centers for Disease Control and Prevention guidance advises that “hospitalized patients with suspected influenza should receive immediate empiric antiviral treatment…, a negative RIDT or DFA test result does not exclude influenza virus infection…” (*10*). Moreover, this guidance also recommends that collection of lower respiratory tract specimens may be useful for reverse transcription–PCR testing to improve diagnosis for patients suspected of having severe lower respiratory tract disease caused by pandemic (H1N1) 2009 virus. The current findings strongly support this recommendation, particularly for severely ill patients.
